# Electronic Excitations in Crystalline Solids through the Maximum Overlap Method

**DOI:** 10.1021/acs.jctc.1c00427

**Published:** 2021-09-30

**Authors:** Loredana Edith Daga, Lorenzo Maschio

**Affiliations:** Dipartimento di Chimica, Università di Torino and NIS Centre, Via P. Giuria 5, 10125 Torino, Italy

## Abstract

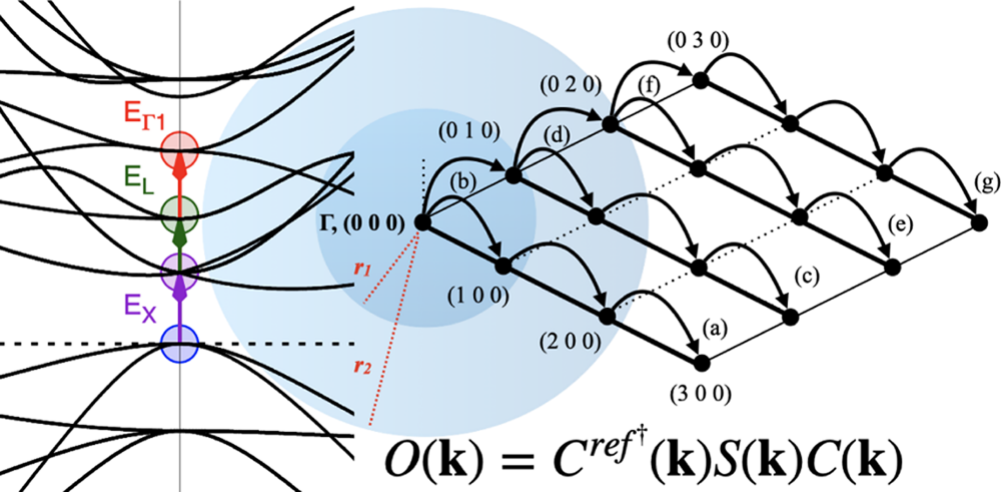

The maximum overlap method (MOM) has emerged from molecular quantum chemistry as a convenient practical procedure for studying excited states. Unlike the Aufbau principle, during self-consistent field (SCF) iterations, the MOM forces orbital occupation to be maximally similar to that of a reference state. Although still within a single-particle framework, this approach allows for the evaluation of excitation energies (Δ-SCF) and geometry optimization of electronic configurations other than the ground state. In this work, we present an extension of the MOM to periodic crystalline solids, within the framework of an atom-centered Gaussian basis set. In order to obtain a realistic concentration of excited electrons, we allow excitation in only one—or a few—points of the Brillouin zone, leading to a fractional occupation of crystalline Kohn–Sham states. Since periodic SCF solution techniques involve an iteration between direct and reciprocal spaces, only totally symmetric excitations are allowed in our treatment, in order to preserve the translational symmetry: vertical Γ-point excitations or collective excitations in a sphere around Γ. Other types of excitations are accessible through folding of the Brillouin zone subsequent to the creation of a supercell. The features and performance of the method are presented through its application to prototypical solids such as bulk silicon, diamond, and lithium fluoride and comparing the results with the available experimental data. The demonstrative application to nickel oxide and solid CuI(piperazine)—a luminescent copper halide compound—highlights the promising potential of the MOM in solid-state quantum chemistry.

## 1 Introduction

Excited states are notoriously much more challenging and costly to be studied through *ab initio methods* than ground states. The most commonly used approaches are many-body methods such as time-dependent density functional theory,^1,2^ configuration interaction singles,^3,4^ and Green’s function of the Bethe–Salpeter equation,^5^ even though a variety of other methods are available.^6^

Such post-SCF and/or multiconfigurational methods are, however, generally demanding, both in terms of computational resources and efforts required for their implementation, and often do not offer many useful tools such as a geometry optimizer or vibrational frequency calculations. Thus, the idea of a simple single-particle approach with the ability to satisfactorily describe excited states by the same methods and tools used for the ground state has a great appeal for routine applications.^7^

In this view, the maximum overlap method (MOM)^8,9^ has seen some success despite its simplicity. Given a reference state, the MOM carries out a standard iterative self-consistent procedure, except that instead of setting orbital occupations according to the lowest energy ranking, it occupies those orbitals with the largest overlap with respect to a reference configuration. In this way, the Aufbau principle is overridden, and the SCF iterations provide the orbitals of the desired electronic configuration much in the same way as for the ground state, thus allowing for the use of the many standard ground-state algorithms such as the gradient calculation for geometry optimization or vibrational frequency calculations. The excitation (or de-excitation) energy can be evaluated simply as the difference between the total energies of the two configurations (Δ-SCF^10−12^). The interest toward MOM-related approaches—and beyond—appears to be alive if not increasing in recent years, and many works have been published on the topic.^13−16^

In this work, we present an extension of the MOM to the case of crystalline solids, treated within periodic boundary conditions (PBCs) using a local atom-centered Gaussian basis set. While such extension might seem trivial at first sight, it poses some conceptual challenges that have to be tackled, due to the periodic nature of the crystal and the features of electronic bands. In particular, (i) since the unit cell is periodically repeated, performing the excitation in direct space would lead to an unrealistically high density of excitations, thus requiring costly supercell calculations as in ref (17); (ii) working in reciprocal space allows for a single electron to be excited within the PBCs, but how can one tune the concentration of excitations? and (iii) evaluation of excitation energies and atomic forces and gradients must properly take into account such concentrations. Moreover, if the iterative SCF procedure—as is the case here—involves going back and forth from reciprocal to direct space through the build of a density matrix, only totally symmetric excitations are allowed in order to preserve the translational symmetry.

As we will show, our solution passes through a fractional occupation of electronic bands (Kohn–Sham states). This is equivalent to thinking in terms of integer occupations of extended generalized Kohn–Sham states in a supercell—what we called “concentration” above. In this connection, we note that fractional occupations in DFT can lead to errors that strongly depend on the amount of delocalization errors of the underlying functional.^18−20^

In the following, we present the simple formalism we developed and discuss its implications in connection with the points listed above through example calculations on simple crystalline systems (Si, diamond, and LiF). We also present demonstrative applications on solid NiO^17^ and CuI(piperazine)^21^ crystals.

## 2 Theory

In this section, starting from the basics of the SCF procedure in periodic systems, we will present the details of the periodic MOM and discuss the consequences of its application to electronic bands. The method has been developed within a local atom-centered (Gaussian) basis set framework^22^ but is generally applicable, for instance, with other approaches such as plane waves or finite-difference grids.

### 2.1 SCF in Periodic Systems

For a crystalline system, Hartree–Fock/Kohn–Sham equations are commonly solved in reciprocal space, in a number *N*_**k**_ of discrete **k**-points constituting a uniform sampling of the first Brillouin zone
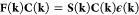
1where, as usual, in each **k**-point, **F**(**k**) is the Fock matrix, **S**(**k**) is the overlap matrix, **C**(**k**) are the coefficients of the crystalline orbitals, and **ϵ**(**k**) are the corresponding eigenvalues. The Fock matrix build is, in turn, carried out in direct space, hence at each iteration a direct-space representation of the density matrix **P** is built from eigenvectors such that
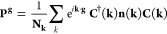
2where **g** is the vector locating a lattice point (cell) in direct space and **n**(**k**) is the occupation matrix, a diagonal matrix with non-null elements in correspondence to the occupied orbitals. In the case of a zero kelvin nonconducting system, which we will assume in this work, such elements are either 1 or 0, and for the ground state occupations are assigned following the Aufbau principle filling in each **k**, the orbitals having the lowest eigenvalues **ϵ**(**k**). From eq 2, it follows that *N*_**k**_ defines the direct space PBCs, that is, the size of the portion of direct space after which the orbital phases are replicated. Hence, the definition of the reciprocal space sampling, which is usually in the hands of the user, directly reflects on the characteristics of the periodic boundaries adopted.

### 2.2 MOM for Periodic Systems

The MOM acts on the definition of the **n**(**k**) occupation matrix of eq 2. Let us start from a reference solution **C**^ref^(**k**)—which can be either from the converged ground state, or an initial guess. Once the eigenvectors are sorted by energy, the code over-rides the Aufbau principle by forcing a different occupation pattern **n**^ref^(**k**). Technically, this can be done in any one—or even more than one—**k**-points of the Brillouin zone, and in the next subsection we will discuss which choices are physically meaningful.

In subsequent iterations, the overlap between the new coefficients **C** and **C**^ref^ is evaluated
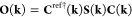
3

The projection of the *j*-th new orbital onto the old occupied space is expressed as
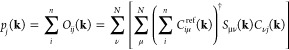
4

For each nonzero diagonal element in **n**^ref^(**k**), the largest corresponding projection *p*(**k**) locates the position to be filled in the new **n**(**k**).

The evaluation of **O**(**k**) as in eq 3 is relatively inexpensive, hence the additional cost of the MOM is virtually negligible with respect to that of the corresponding ground-state method (i.e., HF and DFT), even though convergence can turn out to be more difficult. Convergence accelerators such as DIIS^23,24^ can normally be used within this framework.

Depending on the definition of **n**^ref^(**k**), the MOM can then be used to converge the SCF toward solutions that are different from the ground state. We will focus on this use of the MOM in the following. A further possibility that we do not explore here is to use the method to stabilize the ground state solution, avoiding the intrusion of unphysical states arising from numerical inaccuracies—such as arising from integral screenings and the subsequent early truncation of lattice Fourier transforms, or instabilities due to the use of diffuse functions within the Ewald sums.^25^ We also note that the reference state **C**^ref^ could be kept constant through the SCF or changed at each iteration shifting the reference to the previous cycle. The latter is the choice we adopted, as we found it to lead more consistently to the desired result.

### 2.3 Excitations in Solids through the MOM

#### 2.3.1 Excitation from a Single **k**-point to Another

**Figure 1:**
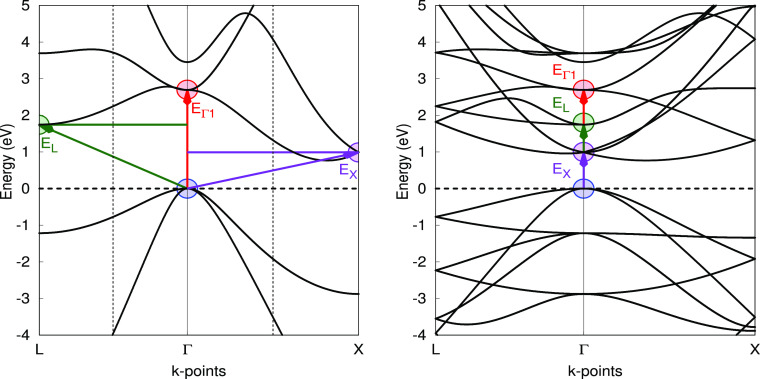
Graphical representation of some possible electronic excitations—labeled as *E*_Γ1_, *E*_*X*_, and *E*_*L*_, as computed in Table 1—in the electronic structure of bulk silicon (PBE functional). Left panel: primitive unit cell. Right panel: 2 × 2 × 2 supercell. Upon folding of the bands in the supercell creation, the excitations *E*_Γ1_, *E*_*X*_, and *E*_*L*_ become all Γ-point-only excitations. The lines along which the band structure is folded are marked by dashed vertical lines in the left panel.

It follows from eq 2 that the translational invariance of the direct space density matrix has to be preserved in the SCF procedure. Subsequently, only excitations that are totally symmetric with respect to the group of lattice translation vectors are possible within our approach. In fact, this property is granted by vertical excitations at the center of the Brillouin zone (Γ-point-only excitations) but neither by vertical excitation in other **k**-points nor by diagonal excitations. Such excitations can, however, be accessed through creation of a supercell—as by increasing the size of the periodically repeating unit in the direct space the reciprocal space folds itself into Γ. In Figure 1, the band structure folding is reported for bulk silicon, for which we report numeric results in the Results section: excitations *E*_*L*_ and *E*_*X*_, which are not accessible through our MOM in the primitive unit cell, both become Γ-point excitations in the 2 × 2 × 2 supercell (right panel).

Vertical Γ-point excitations result in a new **n**^ref^(**Γ**) in which one non-null element has been set to zero and a null element has been set to one. A small variation of the density matrix of eq 2 will follow, leading to a new density **P**_exc_^**g**^. Formally, this excitation results in a fractional occupation of both the bands from which the electron is removed and the one it is excited to. Such fractional occupation is numerically 1/*N*_**k**_. Physically, this is equivalent to having only one single excitation within the periodic boundaries, that is, in a supercell made of *N*_**k**_ unit cells. Hence, the excitation is diluted in the whole PBC, and since the unit cell total energy is computed, the excitation energy for a full electron must be evaluated by multiplying the excitation energy per cell by the inverse of the fractional occupation number 1/*N*_**k**_
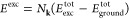
5where *E*_exc_^tot^ is the unit cell energy obtained through **P**_exc_^**g**^. In fact, our reciprocal space MOM allows for a diluted excitation using just the small primitive unit cell—hence with a computationally cheap calculation—as opposite to direct-space Δ-SCF as in ref (17) that also requires a more costly supercell calculation for Γ-point excitations.

#### 2.3.2 Tuning the Concentration of Excited Electrons

It follows from the above discussion that changing the **k**-point sampling also has an impact on the concentration of excited electrons in direct space. It is also possible within our approach to consider an excitation involving not only the Γ-point electron but also a portion of the corresponding valence and conduction bands corresponding to a sphere of radius **r** around Γ. This corresponds to a physical process in which a light that is not precisely monochromatic—hence with some frequency broadening—is used to induce the excitation.

**Figure 2:**
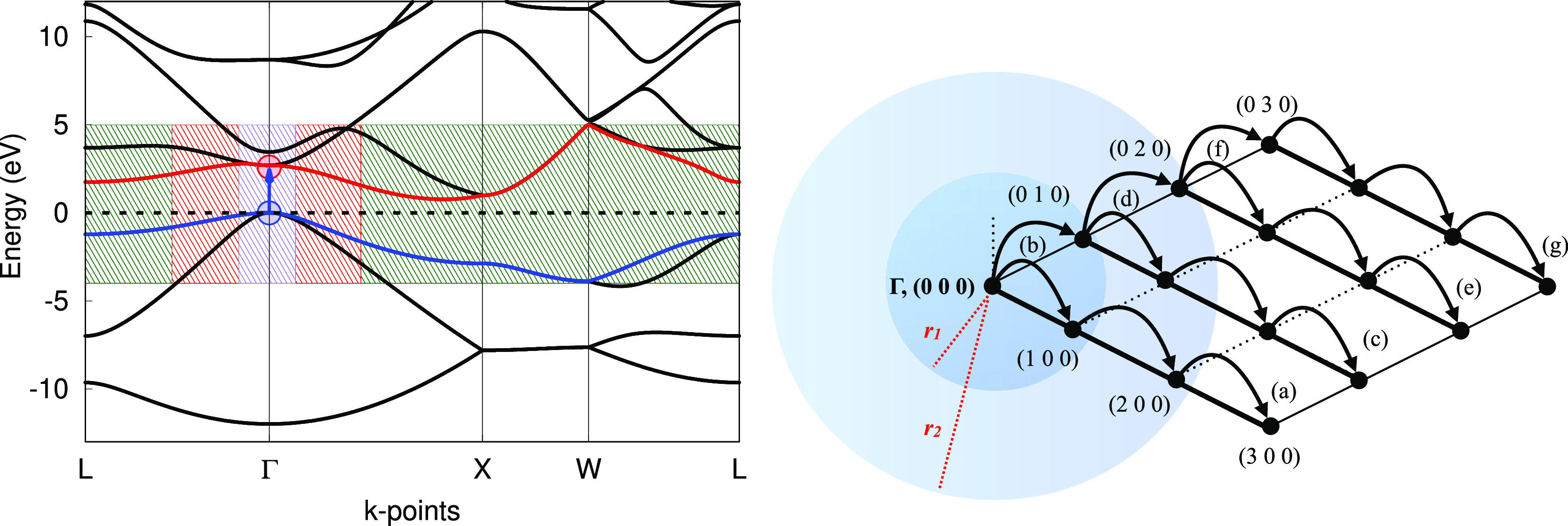
On the left, an excitation in Si bulk (PBE functional), in which the radial-sphere approach is graphically represented. On the right, the path in a 2D Brillouin zone followed to trace the Γ-point bands and the spheres around Γ are represented.

As a first thing, when defining the initial reference excited state, we need to trace the involved bands across the Brillouin zone to cope with possible band crossings and degeneracies. Such approach is graphically described in Figure 2: once the Γ-point excitation is defined, we evaluate the overlap *O*_**k**,**k**′_ between the band eigenvectors in two neighboring points **k** and **k**′, expressed as
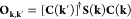
6

The largest overlap elements allow tracing the bands between **k** and **k**′. Since the space we deal with is—in the general case—a 3D one (although all of our approach works for 2D and 1D periodic systems as well), we follow a path in reciprocal space as depicted in the right panel of Figure 2, following subsequent rows starting from Γ until completing the whole grid. Once the tracing is completed, a sphere is defined around Γ, and only the number of **n**(**k**) corresponding to the number of *N*_**k**_^exc^**k**-points is enclosed within this sphere. The excitation defined in Γ is also then performed in these points according to the band tracing information. Since the excitation zone is spherical around Γ, the total symmetric character of the density **P**_exc_^bfg^ is preserved and then it can be effectively represented in direct space (i.e., the total density remains periodic in the 1 × 1 × 1 unit cell). Experimentally, increasing the radius of such sphere corresponds to (1) a broadened (non-exactly monochromatic) light triggering the excitation and (2) a higher density of excited electrons, which are now *N*_**k**_/*N*_**k**_^exc^ within the PBC. The fractional occupation of the excitation band is, in fact, *N*_**k**_^exc^/*N*_**k**_

The energy of the excitation is then evaluated as
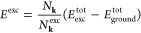
7

### 2.4 Energy Gradients and Geometry Optimization

Let us start from eq 5 and sum back the SCF total energy of the ground state to obtain the unit cell total energy of the MOM excited state
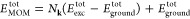
8

Note how *E*_MOM_^tot^ is different from the SCF output energy *E*_exc_^tot^, which contains a fractional excitation. If the excitation is not restricted to Γ, eq 7 should be used instead. Taking the derivative of eq 8 with respect to atomic displacements, we obtain
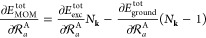
9where  is the coordinate of atom A along a general Cartesian direction *a*. Analogous equations hold for cell gradients, which can always be expressed in the form of atomic gradients.^26^

During a geometry optimization procedure, at each geometry, the ground- and excited-state gradients are needed for the evaluation of eq 9, thus requiring two SCF procedures.

## 3 Results

In this section, we present some demonstrative calculations using our MOM, with the purpose of validating the approach and showing its capabilities. To this aim, we have tested the lowest-energy excitations in a small group of simple solids including LiF (ionic crystal), Si (covalent semiconductor), and diamond (covalent insulator). In addition, we have investigated two cases with more applicative potential, namely, NiO and the solid CuI-piperazine. All calculations were performed with a development version of the CRYSTAL program.^22^

We have adopted triple-ζ electron basis sets from Peintinger et al.^27^ for Si, C, and LiF. We applied the same basis sets for the CuI-piperazine, while in the NiO application, a dcm-tzvp^28^ basis has been used.

### 3.1 Excitation Energies

In Table 1, we report Γ-point excitations as computed with the MOM and compare the results with experiments available from the literature. As it is more than well known,^29,30^ the main impact of the functional choice on the electronic structure is on its band gap, and the amount of exact exchange plays a major role in that. As already reported in literature for the fundamental band gap,^31,32^ the range-separated HSE06 functional proved to provide a successful balance in that, and this is observed in our results for covalent crystals also, where it consistently leads to excitation energies within 0.1 eV from experimental references for silicon and diamond. However, for LiF, PBE0 seems to represent a better approximation. The difference between the triplet and singlet excited states is always in favor of the latter, which lays in all three cases at a lower energy. The difference is strongly dependent on the amount of exact exchange included, thus suggesting a role of excitonic effects. In this connection, we also remind the reader about the relationship between the delocalization error and the fractional occupation discussed in the Introduction. As per the nature of our single-determinant approach, we use here the terminology “singlet” and “triplet” to briefly indicate parallel and antiparallel spins.

**Table 1:** Excitation Energies (in eV) for Simple Solids as Computed with the MOM with Different Functionals^a^

	method	*E*_Γ_^sing^	*E*_Γ_^trip^	*E*_*X*_^sing^	*E*_*L*_^sing^
silicon	Exp.	3.4^33−35^		1.2^34^	2.0^34^
		3.45^36^			
	PBE	2.691	2.688	0.988	1.743
	HSE06	3.422	3.416	1.577	2.440
	PBE0	4.048	3.993	2.162	3.416
					
diamond	Exp.	6.0^37,38^		5.46–5.6^34^	
		7.75^35^			
	PBE	5.619	5.618	4.795	
	HSE06	7.000	6.988	5.942	
	PBE0	7.677	7.598	6.552	
					
LiF	Exp.	12.6^39,40^			
	PBE	8.993	8.992		
	HSE06	11.300	11.289		
	PBE0	12.089	11.859		

a A primitive cell (no supercell) is used for Γ-point excitations (*E*_Γ_^sing^ and *E*_Γ_^trip^). A 2 × 2 × 2 supercell has been adopted for *E*_*X*_^sing^ and *E*_*L*_^sing^. Available experimental values from the literature are reported for each system.

**Figure 3:**
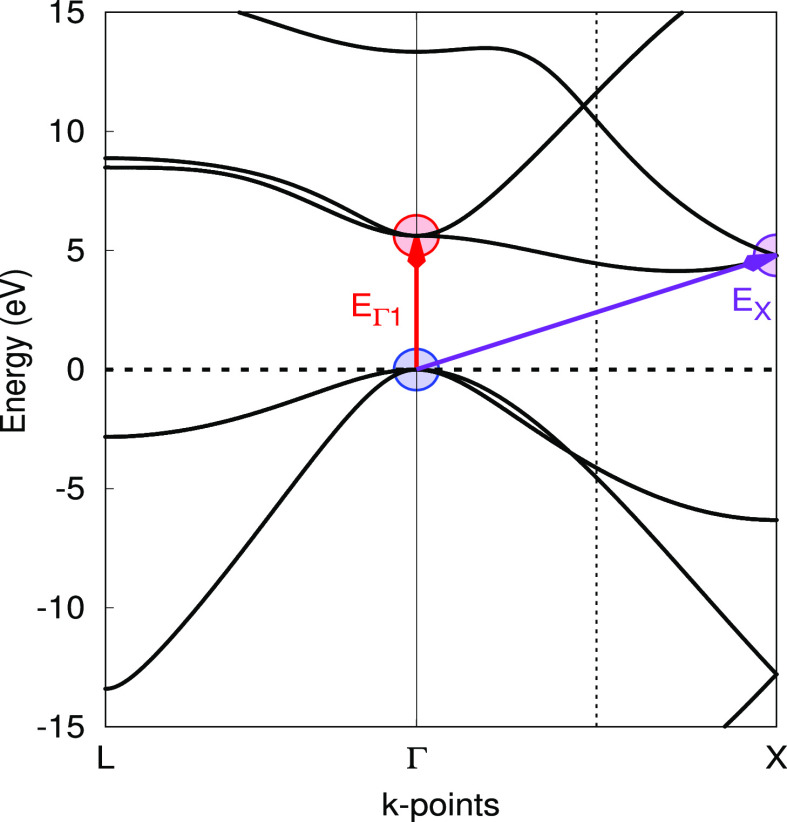
Diamond band structure of the primitive unit cell calculated using the PBE functional.

The first two columns of Table 1 can be obtained with a primitive cell or a supercell, yielding exactly the same results. The *E*_*X*_ and *E*_*L*_ columns, however, were obtained adopting a 2 × 2 × 2 supercell that allows the bands in *X* and *L* points to fold in Γ, as shown in Figures 1 and 3. The reciprocal space grid was reduced to 4 × 4 × 4 for consistency.

As discussed in the theory section, the density of excited electrons can be tuned either by changing the **k**-point sampling of the Brillouin zone or by exciting *N*_**k**_^exc^**k**-points within a sphere of radius *r*_s_ around the Γ point (see Figure 2) The tuning of the number of *N*_**k**_ points and the radius *r*_s_ allows assessing any desired exciton density.

In Table 2, we show the combined effect of the two parameters in the case of bulk silicon:

- Increasing the radius *r*_s_ rapidly increases the number of points enclosed in the sphere. As a consequence, the excitation energy becomes higher due to the increased concentration of excited electrons
- In the second series of data in Table 2, we show that, by changing both parameters simultaneously, we can keep *N*_**k**_^exc^ constant while increasing the size of the PBC volume. The excitation energy decreases until a dilution comparable to the [*N*_**k**_ = 512; *r*_s_ = 0.01] case is reached.

**Table 2:** Singlet Excitation Energies (in eV) for Bulk Silicon (PBE Functional) as Functions of the Number of Points Sampling the Brillouin Zone (*N*_**k**_) and the Radius of the **k**-point Sphere around Γ (*r*_s_)^a^

*N*_**k**_	*r*_s_	*N*_**k**_^exc^	*E*^exc^
512	0.01	1	2.691
512	0.018	9	4.566
512	0.025	15	4.893
512	0.05	21	5.648
512	0.08	47	5.587
512	0.1	53	6.110
512	0.3	247	9.227
			
512	0.025	15	4.893
1728	0.015	15	2.972
4096	0.010	15	2.794
5832	0.008	15	2.744
13,824	0.005	15	2.621

a *N*_**k**_^exc^ indicates the number of points enclosed in the sphere having radius *r*_s_.

By progressively increasing *r*_s_, we can reach a point in which the whole valence band is excited to the whole conduction band, that is, one electron per unit cell is excited. Such a situation is barely physical, especially in covalently bonded semiconductors. The excitation energy becomes, in fact, extremely high. In the case of LiF (not shown here), excitations are localized and then the penalty due to a high density of excitons is smaller because they interact little one with another.

### 3.2 Geometry Optimization and Luminescence

#### 3.2.1 Nickel Oxide

Even though nickel oxide is quite a well-known material and has a simple atomic structure, its magnetic and electronic properties making it a very interesting system. In a recent work,^17^ its excited-state structure has been studied extensively through Δ-SCF methods within the Crystal code but with an approach different from the MOM. In short, the approach in ref (17) consists of forcing an excited state through atomic orbital occupations in the initial guess and eigenvalue shifting. This is in practice a direct space approach that leads to the excitation of entire bands (across the whole BZ)—hence a supercell calculation is mandatory in order to reach a realistic dilution of excitation. Reference (17) testifies how a detailed analysis of the excitations in NiO must be carried out with great care, given the magnetic phases possible, the number of relevant excited states, as well as the delicate role of functional and basis set choice.

Such a detailed study goes well beyond the scope of this work. Our aim here is to validate our method on a system that is somewhat more complex than those in Table 1 and to test our excited-state optimization algorithm. NiO, in fact, possesses excited states that live long enough to give rise to observable Stokes shifts. We considered here only the ferromagnetic (FM) phase of NiO, which lends itself well to our purpose because due to its simple structure there are no internal degrees of freedom, so that only the lattice parameter is subject to optimization. Even considering that NiO is antiferromagnetic in nature, we believe the FM phases well serve our validation purposes.

**Figure 4:**
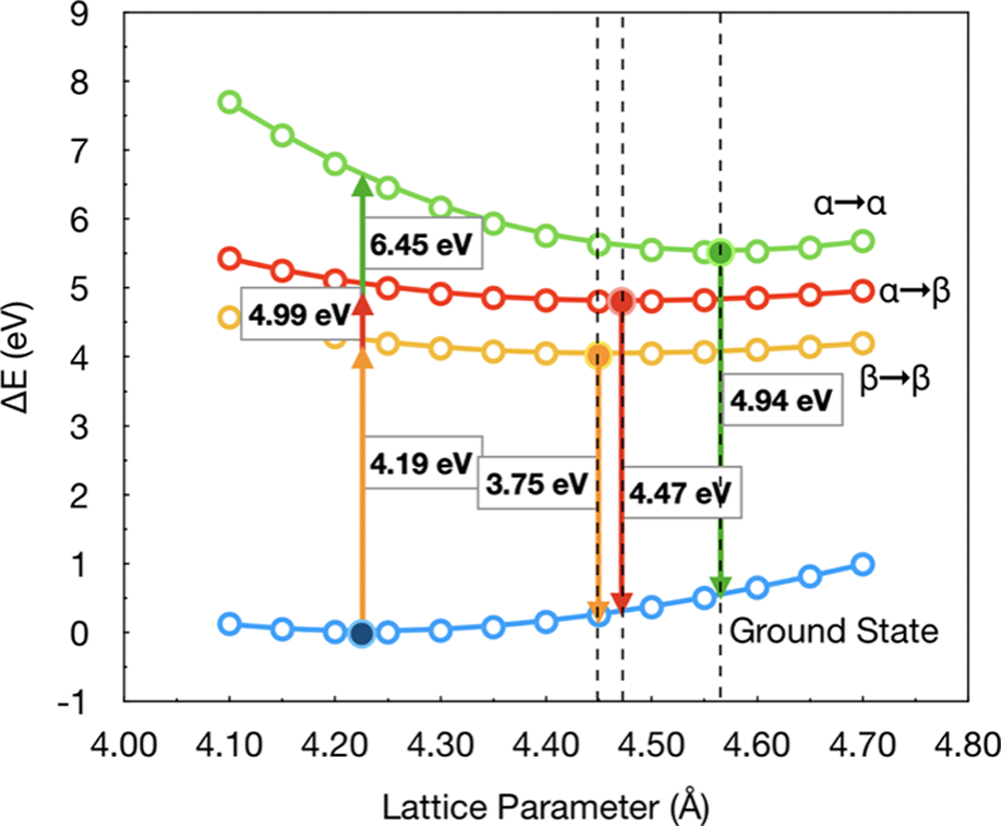
Energy of the ground and first excited states of bulk ferromagnetic NiO as a function of lattice parameter. The ground-state minimum is taken as a reference (Δ*E* = 0). The B3LYP functional was used. The full bullets mark the results of the geometry optimizations using analytical gradients as in eq 9.

In Figure 4, we present our results. We have considered the three lowest excited states in the Γ point—note that NiO has an indirect band gap, so these excitations do not correspond to the band gap—supercells would be needed to reach other parts of the Brillouin zone. Two of such excitations are labeled α → α and β → β, that is, α and β highest occupied molecular orbital (HOMO)–lowest unoccupied molecular orbital (LUMO) transitions (in our ferromagnetic phase, there are 19 electrons in α bands and 17 in β ones). The third is the spin-flip excitation from α-HOMO to β-LUMO. These correspond in Figure 4 to green, yellow, and red curves, respectively.

For each of the above-listed states, we have run both a geometry optimization with analytical gradients and a series of single-point calculations. The results clearly show that our MOM gradients correctly find the right minimum of the excited state curves in all cases, located at 4.45, 4.47, and 4.56 Å. The Stokes shifts are 0.44, 0.5, and 1.51 eV. As a final note, we remark that only a calculation on the primitive unit cell was needed using our MOM, while a reciprocal space grid of 8 × 8 × 8 points was used, hence describing the excitation of one electron every 512 unit cells.

#### 3.2.2 Solid CuI(Piperazine)

Among the luminescent copper(I) halides, [CuI(piperazine)_0.5_]_*∞*_ is a peculiar compound that exhibits dual luminescence, a feature that is of potential relevance in technological applications. In recent years, within the framework of a synergetic theoretical–experimental study,^21^ we have characterized its excitations through *ab initio* post-SCF methods.^41^ At that time, we were not able to investigate the actual luminescence properties, as we had no tools for optimizing geometries in the excited state, as we have developed in this work.

We here apply our MOM geometry optimizer to this structure so as to analyze the structural and electronic changes of the long-lived excited state. As in previous work,^21^ we considered two excitations around the Fermi level, namely, HOMO → LUMO and HOMO – 1 → LUMO + 1, in the Γ-point only. A pob-TZVP basis set was used, along with a hybrid PBE functional with 10% of HF exchange.

**Figure 5:**
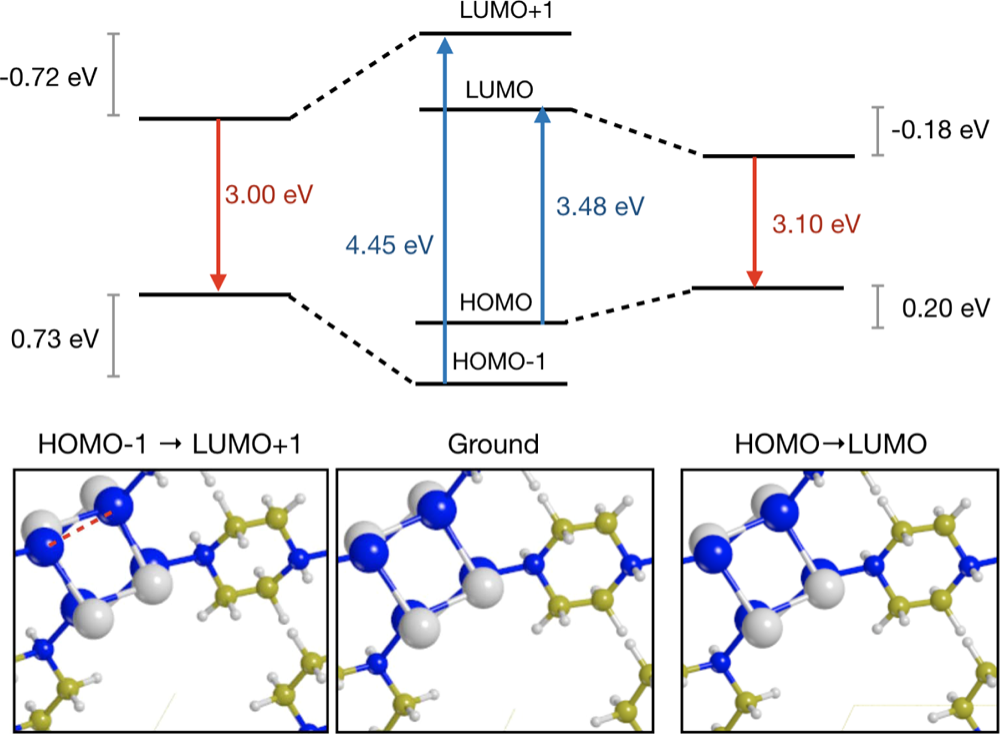
Energy levels and atomic structure of solid CuI-piperazine around the Fermi level at the ground-state geometry (center) and at the geometries optimized after HOMO → LUMO (right) and HOMO – 1 → LUMO + 1 (left) electronic excitations.

The main results of our MOM calculations are reported in Figure 5 and Table 3. From the figures, it is seen that the structural relaxation of the excited states leads to mild but significant modifications, mostly seen in the rotation of the organic ring. From Table 3, we can also see that the Cu–Cu distance is reduced up to 4% in the highest excitation, and most notably that the cell parameters undergo quite a change, especially in the HOMO → LUMO excitation, which results in a volume expansion. In the HOMO – 1 → LUMO + 1 case, the volume does not change so significantly, but a cell distorsion is observed, with an elongation along the *c* axis.

**Table 3:** Cu–Cu Distance and Cell Parameters (All in Å) for the Structures of Solid CuI Piperazine Optimized for the Ground and Excited States

	ground state	HOMO → LUMO	HOMO – 1 → LUMO + 1
Cu–Cu distance	3.517	3.483	3.384
*a*, *b* cell parameters	9.499	9.650	9.503
*c* cell parameter	6.774	6.805	7.158

The effects of the geometry relaxation on the luminescence energies are more pronounced on the highest excitation than on the lowest, with the results of the two corresponding emissions being 3.0 and 3.1 eV, respectively. This result is in qualitative agreement with the results of Figure S3 in the Supporting Information of ref (21), which shows that two excitations exist with a markedly different excitation energy but similar emission. Quantitatively, our excitation energies are in reasonable agreement, while the emission energy is evidently still too large with respect to the experiment. A more detailed study would be needed to clarify this, with a careful analysis of the role of basis sets and functional, which goes beyond the purpose of this paper.

## 4 Conclusions

In this work, we have presented a periodic implementation of the MOM. It allows selecting electronic excitations and optimizing the geometries of excited states while keeping a computationally cheap SCF approach as used for the ground state. Due to the iterative transitions from direct to reciprocal space and back, our approach works with excitations that preserve the totally symmetric nature of the electron density, namely, Γ-point excitations or collective excitations in a sphere of **k**-points around Γ. A calculation using the primitive unit cell allows describing the excitation of only one electron within the PBCs, avoiding costly supercell calculations. Such a supercell approach is, however, needed to access excitations far from the center of the Brillouin zone.

Through demonstrative applications, we have shown how the MOM can be easily applied to a variety of crystalline solids, from prototypical simple crystals to complex organic–inorganic frameworks, with full control on the electronic occupations and spins.

As a future perspective, we plan to implement vibrational frequencies and a representation of electronic densities, which would significantly extend the usefulness and applicability of the approach.
